# Elevated Serum Melatonin under Constant Darkness Enhances Neural Repair in Spinal Cord Injury through Regulation of Circadian Clock Proteins Expression

**DOI:** 10.3390/jcm8020135

**Published:** 2019-01-23

**Authors:** Yunkyung Hong, Yunho Jin, Kanghui Park, Jeonghyun Choi, Hyunbon Kang, Sang-Rae Lee, Yonggeun Hong

**Affiliations:** 1 Department of Physical Therapy, College of Healthcare Medical Science & Engineering, Inje University, Gimhae 50834, Korea; dangmoo777@naver.com; 2 Department of Rehabilitation Science, Graduate School of Inje University, Gimhae 50834, Korea; jynh33@naver.com (Y.J.); yiopiop0011@nate.com (J.C.); 3 Ubiquitous Healthcare & Anti-aging Research Center (u-HARC), Inje University, Gimhae 50834, Korea; jspt95@hanmail.net (K.P.); rkdguasqhs23@naver.com (H.K.); 4 Biohealth Products Research Center (BPRC), Inje University, Gimhae 50834, Korea; 5 Department of Physical Therapy, Dong-Ju College, Busan 49318, Korea; 6 Department of Physical Therapy, Graduate School of Inje University, Gimhae 50834, Korea; 7 National Primate Research Center (NPRC), Korea Research Institute of Bioscience and Biotechnology (KRIBB), Ochang 28116, Korea; srlee@kribb.re.kr

**Keywords:** environmental lighting condition, endogenous melatonin, spinal cord injury, circadian clock, neural remodeling

## Abstract

We investigated the effects of environmental lighting conditions regulating endogenous melatonin production on neural repair, following experimental spinal cord injury (SCI). Rats were divided into three groups randomly: the SCI + L/D (12/12-h light/dark), SCI + LL (24-h constant light), and SCI + DD (24-h constant dark) groups. Controlled light/dark cycle was pre-applied 2 weeks before induction of spinal cord injury. There was a significant increase in motor recovery as well as body weight from postoperative day (POD) 7 under constant darkness. However, spontaneous elevation of endogenous melatonin in cerebrospinal fluid was seen at POD 3 in all of the SCI rats, which was enhanced in SCI + DD group. Augmented melatonin concentration under constant dark condition resulted in facilitation of neuronal differentiation as well as inhibition of primary cell death. In the rostrocaudal region, elevated endogenous melatonin concentration promoted neural remodeling in acute phase including oligodendrogenesis, excitatory synaptic formation, and axonal outgrowth. The changes were mediated via NAS-TrkB-AKT/ERK signal transduction co-regulated by the circadian clock mechanism, leading to rapid motor recovery. In contrast, exposure to constant light exacerbated the inflammatory responses and neuroglial loss. These results suggest that light/dark control in the acute phase might be a considerable environmental factor for a favorable prognosis after SCI.

## 1. Introduction

Spinal cord injury (SCI) is a type of traumatic disorder of the central nervous system (CNS) that causes severe physical disability accompanied by neurological problems [1]. Injury-induced neural damage occurs throughout the entire spinal cord, including the primary lesion site. Mechanical trauma primarily kills either neurons or glial cells in the injury epicenter, and then insidious secondary damage follows in the penumbra. The latter is mediated by intracellular injurious signaling molecules that form a deleterious network, which can persist for several years [2]. Consequently, activated neuroinflammatory responses spread into the adjacent region, leading not only to surrounding edema but also to increased permeability of the blood-CNS barrier [3]. Upper motor neurons starting from the brain project to lower motor neurons in the spinal cord. SCI means that the signal from the brain cannot be conveyed via upper to lower motor neurons, leading to muscle paralysis below the injury level [4]. Since SCI may accompany these pathologic states leading to physical disability and mortality, effective care to minimize neural damage for favorable prognosis should be required.

Neuronal-glial remodeling processes were also observed in the whole spinal cord following SCI. Interventional approaches have aimed to promote the reorganization of the disrupted neural circuit. Transplantation of neural stem cells to the injured spinal cord may be promising, but it might be followed by biological cost, including immune suppression [5,6]. Induced pluripotent stem cell-derived cell therapy may not induce immune rejection responses [7], but this invasive method requires some care after the implementation. Manipulation of endogenous neural stem cells might become a cue for these problems. In response to SCI, endogenous neural stem cells are differentiated to fill in the vicinity of the destructive lesion mostly with neuroglia [8]. Neuronal differentiation is extremely limited by the microenvironment in the traumatic zone [9]. Instead, surviving intrinsic spinal neurons distributed across peri-lesion sites are reconnected to compensate for the behavioral defect [10]. Previously, it was reported that melatonin treatment combined with exercise increases not only neurite outgrowth but also dendritic spine density after SCI [11]. 

The first studies on the beneficial effects of melatonin (N-acetyl-5-methoxytryptamine) were published approximately three decades ago. By the early 2000s, evaluations of melatonin efficacy have been expanded into animal models with CNS damage [12,13,14]. Numerous studies have reported that melatonin exerts the neuroprotective effects on SCI animals through anti-inflammatory and anti-oxidative intracellular signals [15,16]. Besides the application of exogenous melatonin, the regulation of endogenous melatonin secretion has been suggested as a feasible intervention for SCI. Environmental lighting is a major factor that impedes nocturnal melatonin secretion [17], and constant light exposure has been shown to postpone functional recovery following SCI in rats [18]. Although it is well-known that melatonin is synthesized more under the lightless condition of nighttime, studies showing the effects of environmental darkness after CNS injury have not been reported to date. Furthermore, the melatonin precursor N-acetylserotonin (NAS) is known to circadian-dependently promote TrkB activation [19]. This precursor is synthesized by the clock-driven enzyme encoded by arylalkylamine N-acetyltransferase gene (*Aanat*) [20]. It has been documented that melatonin regulates the pro-survival mechanism via TrkB-induced Akt/ERK signaling [21]. Although it is well known that melatonin is synthesized more under the lightless condition of nighttime, studies showing the effects of environmental darkness after CNS injury have not yet been reported. According to our previous study, melatonin may contribute to the neural recovery after SCI [17,22]. In this context, controlling endogenous melatonin secretion would be a therapeutic option for SCI treatment. Therefore, whether environmental light/dark condition affects early recovery from CNS injury should be elucidated. In the present study, we demonstrated that conditional controlled light/dark differentially influences the circulating melatonin level, neuronal-glial cell fate in the lesion site, and compensatory plasticity in the adjacent core lesion. Therefore, we suggest that environmental light/dark control might be important in especially acute SCI care.

## 2. Materials and Methods

### 2.1. Experimental Groups

**Figure 1:**
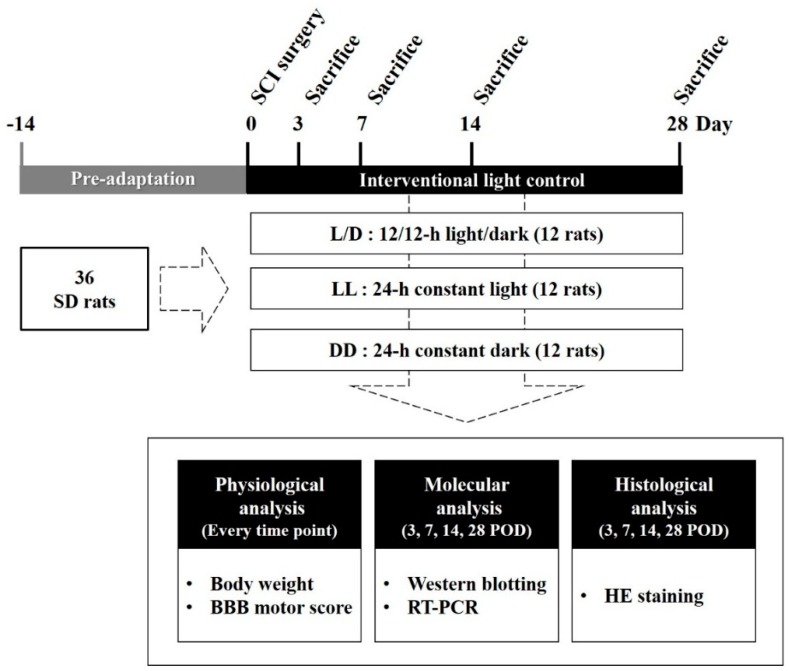
Experimental scheme of the study. Experimental animals were randomly divided into 3 groups: 12/12-h light/dark (L/D), 24-h constant light (LL), and 24-h constant dark (DD). The animals were sacrificed 3, 7, 14, and 28 days after injury for further analyses.

All animal procedures were approved and performed in accordance with the guidelines and protocols approved by the Institutional Animal Care and Use Committee of Inje University (approval No. 2015-05). A total of 36 male Sprague–Dawley rats (SD rats; 8-week-old; 250–280 g) were used. We applied the different light/dark conditions to alter the melatonin biosynthetic pathway through regulation of arylalkylamine N-acetyltransferase (AANAT) expression [22]. Conditional controlled light/dark cycle was applied as follows; 12/12-h light/dark (L/D), 24-h constant light (LL), and 24-h constant dark (DD). ZT0 was set at 07:00 in the L/D condition. The rats were pre-adapted for at least 2 weeks prior to SCI. After the pre-adaptation, animals were randomly divided into 3 groups; L/D, LL, and DD. In turn, each group consisted of 12 rats which were recategorized by 4 sacrifice points (POD 3, 7, 14, and 28). In this way, 3 rats per group were assigned to each time point (Figure 1). At each sacrifice point, CSF was collected, then, the animals were sacrificed for further analyses. For the analyses, we collected both the primary lesion site (T9–11) and indirect injured segments (T6–8, L1–2).

### 2.2. Surgical Procedures for Experimental SCI

Experimental animals were anesthetized with 3% isoflurane in a mixture of 30% O_2_ and 70% N_2_O by mechanical ventilation with an anesthesia system (Harvard Apparatus Inc., Holliston, MA, USA). Induction of inhalation anesthesia was applied for at least 3 min. The anesthesia was maintained with 1% isoflurane after observing significant changes in the heart rates, respiratory rate, and thoracic movements. The thoracic areas of anesthetized rats were exposed after shaving, and then alcohol was applied to sterilize the skin. The paraspinal muscles and tissue adjacent to the vertebrae were dissected to clarify the position of the vertebral column. Laminectomy was performed at the T10 segmental level to expose the dorsal surface of the spinal cord. A modified New York University-Multicenter Animal Spinal Cord Injury Study (NYU-MASCIS) weight-drop device was applied for SCI. A 10 g weight rod was allowed to drop from a height of 25 mm (=0.098 N) onto the dorsal surface of the dura matter [23].

### 2.3. Assessment of Motor Function

Motor function was assessed using the Basso, Beattie, and Bresnahan (BBB) locomotor scale, the scores of which range from 0 (no spontaneous locomotor activity) to 21 (normal coordinated movements) [24]. At least two inspectors participated in the behavioral assessment on PODs 3, 7, 14, and 28. Scores from 0 to 7 indicate isolated spontaneous movements in the hip, knee, and ankle joints. Scores from 8 to 13 indicate well placement of the paw as well as coordinated movements with forelimbs. Scores from 14 to 21 represent toe clearance during stepping, a predominant paw position, trunk stability, and the tail position. The BBB score measurement was performed at 7:00 pm of each test day since once the animals had had congenital nocturnal circadian clock, that time was considered to be optimal in rodent. As general criteria, any animals showing no normal movement before being subjected to the surgery were not considered physically intact, and excluded from the experiment. Similarly, no complete paralysis right after SCI surgery were considered as failed SCI induction following inappropriate rod drop on their spinal cord (<0.098 N), and these animals were excluded from experiment.

### 2.4. Measurement of Melatonin Concentration in Cerebrospinal Fluid

Cerebrospinal fluid (CSF) samples were collected from the cisterna magna at ZT13-ZT15. Since repeated CSF collection might alter the physiological state of the animals, CSF was obtained right before the sacrifice. Collected CSF samples were centrifuged at 2000× *g* for 10 min and stored at −80 °C until further analysis. The samples were thawed and run in at least triplicate. The melatonin levels were quantified using commercially available enzyme-linked immunosorbent assay kits (Cloud-Clone Corp., Houston, TX, USA). Immunoassay was performed using the Fluorescence Multi-Detection Reader (BIOTEK, Winooski, VT, USA) at an absorbance of 450 nm. The concentration of melatonin was quantified using the GraphPad PRISM 5.0 program (GraphPad Software, La Jolla, CA, USA). A nonlinear regression analysis was used to derive an equation to predict the concentration of the unknown samples.

### 2.5. RNA Isolation and Quantificative RT-PCR

The total RNA in each segment was isolated with TRI Reagent (Sigma-Aldrich, St. Louis, MO, USA). The concentration of RNA was determined using a spectrophotometer (Mecasys, Daejeon, Korea). RNA (1 μg) was reverse transcribed using reverse transcriptase (Invitrogen, Carlsbad, CA, USA). The cDNA was amplified with specific primers (Table 1) [25]. Quantitative PCR was performed using a LightCycler 1.5 system (Roche Instrument Center AG, Rotkreuz, Switzerland) with LightCycler FastStart DNA Master SYBR Green I. Controls consisting of double-distilled H_2_O were negative for the target and housekeeper genes. The cDNA samples (2 μL for a total volume of 20 μL per reaction) were analyzed in the same reaction. The cycle temperatures and times followed the manufacturer’s protocol. Relative changes in gene expression were assessed by the delta–delta CT method. Each sample was assessed at least in duplicate.

### 2.6. Protein Extraction and Western Blotting

The spinal segments were lysed in buffer (50 mM Tris-HCl (pH 7.4), 150 mM NaCl, 1 mM NaF, 1 mM Na_3_VO_4_, 1% NP-40, 0.25% sodium deoxycholate, and protease inhibitor cocktail; Roche diagnostics GmbH, Mannheim, Germany). The lysates were incubated on ice and then centrifuged at 4 °C. After collecting the supernatant, the protein concentration was measured using the Bradford protein assay (Bio-Rad Laboratories, Hercules, CA, USA). The proteins were separated by sodium dodecyl sulfate-polyacrylamide gel electrophoresis and transferred to polyvinylidene fluoride membranes (Merck Millipore, Billerica, MA, USA). The membranes were blocked with 5% non-fat milk in TBS with Tween-20 (TBST). Blocked membranes were incubated with primary antibodies with the dilution range of 1:200–1:1000: Bax, Bcl-2, BDNF, BMAL1, Catalase, CKIε, CLOCK, DCX, ERK, p-ERK, MT1, MT2, Nestin, Olig2, PER1, Tau, and GAPDH (Santa Cruz Biotechnology, Santa Cruz, CA, USA); AKT, p-AKT, cleaved caspase-3 (cCasp3), GFAP, nNOS, TrkB, Vimentin (VIM) (Cell Signaling Technology, Danvers, MA, USA); NeuN (Merck Millipore, Billerica, MA, USA); MAP2, PSD-95 (Abcam, Cambridge, UK). Consequently, samples were incubated with secondary antibodies with concentration of 1:5000–1:20,000. Then, the secondary antibody-bound membranes were washed with TBST. Membranes were incubated with the manufacturer-instructed concentration of enhanced chemiluminescence (ECL) reagents (Thermo Scientific, Waltham, MA, USA). ECL-treated blots were immediately exposed to X-ray film (Fuji, Kanagawa, Japan) and quantified using Image J ver. 1.6 (NIH, Bethesda, MD, USA).

### 2.7. Hematoxylin and Eosin Staining

Transcardial perfusion and fixation with 4% neutral buffered paraformaldehyde (NBP; pH 7.4) was conducted. Spinal cord samples were embedded in OCT compound, and then serial horizontal sections (10 μm) were made using a cryostat microtome (MICROM International GmbH, Walldorf, Germany). Sections were stained with hematoxylin and eosin (Sigma-Aldrich) for general morphology, observed under an Olympus DP70 microscope with digital camera (Olympus, Tokyo, Japan) connected to a computer, and then photographed at ×4 and ×20 objective magnifications.

### 2.8. Golgi-Cox Staining and Sholl Analysis

The injured spinal cord segments of each group were cut and transferred to a vial containing solution provided by the FD Rapid GolgiStain kit (FD NeuroTechnologies, Ellicott City, MD, USA. The spinal cord tissues were then immersed in silver impregnation solution for 2 weeks in the dark, followed by immersion in Solution C for a week. The stained tissues were serially sectioned by 100 μm-thickness using vibratome, and then mounted on gelatin-coated slides. Sections were dried up for 1 day at room temperature followed by staining Solution D and E. Then the sections on slide were serially dehydrated from low to high concentration of ethanol. Next, the slides were clarified using xylene before coverslipping with Permount (Fisher Scientific, Pittsburgh, PA, USA). After coverslipping, the sectioned tissues were investigated using OLYMPUS DP70 microscope with digital camera (Olympus, Tokyo, Japan) connected to a computer. Sholl analysis was performed using the NIH Image J Sholl analysis Plugin. Background dendrites extending into the image view from neighboring neurons were manually deleted. The origin of the concentric radii was set at the midpoint of the longest axis of the soma. The number of dendritic branches was counted and visualized by different colors; warmer hues indicate higher number of dendritic branches. Analysis parameters were as follows: starting radius, 1 μm; ending radius, 500 μm; radius step size, 20 μm. Statistical analyses were performed using the SPSS ver. 23.0 (IBM, New York, NY, USA).

### 2.9. Statistical Analysis

Data were collected from repeated experiments and are presented as the mean ± standard deviation (SD) in graphs. The changes in body weight, melatonin level, and behavioral data were analyzed with repeated-measure ANOVA and Tukey’s post-hoc comparison. Statistically significant molecular changes between the groups were assessed using one-way analysis of variance (ANOVA) with Tukey’s post-hoc test. Differences were deemed statistically significant at a *p*-value less than 0.05. All analyses were performed using the statistical software SPSS ver. 23.0 (IBM, Armonk, NY, USA).

## 3. Results

### 3.1. Time-Dependent Changes in Body Weight, Motor Function, and Melatonin Levels

**Figure 2:**
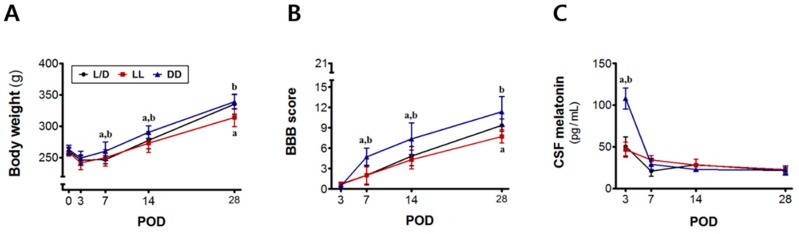
Spontaneous motor recovery of spinal cord injured animals and time-dependent changes of endogenous cerebrospinal fluid (CSF) melatonin. (**A**) There were significant differences in the changes of body weight among the spinal cord injury (SCI) groups were observed. Animals caged in the constant dark condition showed greater body weight from postoperative days (POD) 7 compared to other groups; (**B**) Spontaneous behavioral recovery was suppressed under LL condition, but DD condition enhanced motor function after the seven postoperative days (POD) compared to natural light/dark cycle; (**C**) Cerebrospinal fluid (CSF) was collected between ZT13 and ZT15 under the light/dark cycle. Endogenous CSF melatonin was more concentrated at POD 3 under DD condition, but the mean value did not differ thereafter. L/D, 12/12-h light/dark; LL, 24-h constant light; DD, 24-h constant dark. ^a^ *p* < 0.05, vs. L/D; ^b^ *p* < 0.05, vs. LL.

To induce changed diurnal cycle of endogenous melatonin synthesis, light/dark cycle was conditionally controlled as following; 12/12-h light/dark (L/D), 24-h constant light (LL), and 24-h constant dark (DD). At 3, 7, 14, and 28 days after SCI surgery, the body weight and functional recovery were assessed, and then the spinal cord tissues were excised from sacrificed animals for further analyses. The mean body weight was greater in the SCI + DD group than that of the L/D and the LL group, which appeared from POD 7 (^a,b^ *p* < 0.05, Figure 2A). The BBB scores gradually increased in all of the spinal cord injured rats with time, indicating spontaneous behavioral recovery regardless of the light/dark condition. A remarkable increase of the behavioral scores from POD 7 was seen in the rats exposed to DD condition (^a,b^ *p* < 0.05), while LL condition delayed the time for locomotor recovery (^a^ *p* < 0.05, Figure 2B). Interestingly, increased CSF melatonin level was found in all animals at POD 3. Especially, rats with constant dark condition showed the greatest CSF melatonin concentration (^a,b^ *p* < 0.05, Figure 2C). This tendency was temporary, and no differences were found in further timepoints.

### 3.2. Elevated Melatonin during Acute Phase Brings Molecular Changes at the Injury Epicenter

**Figure 3:**
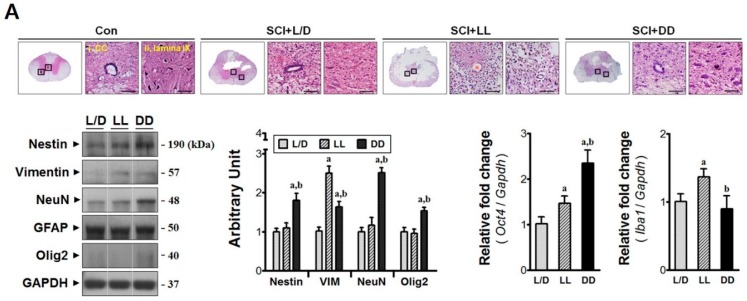

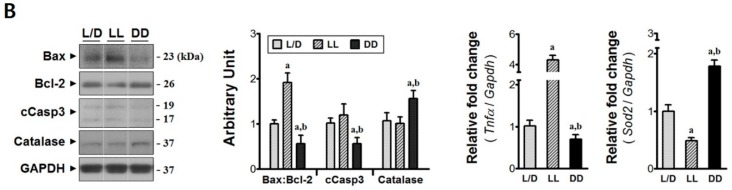
The neuroprotective effects of elevated melatonin on the primary injury region through regulation of anti-inflammatory and anti-oxidant genes. The damaged segments of spinal cord (T9-11) were analyzed at POD 3. (**A**) LL condition exacerbated the histological deterioration of the primary lesion, where *Iba1* expression (microglial marker) was upregulated. DD condition not only protected injured tissue but also increased the levels of endogenous pluripotency markers (Nestin, Vimentin, *Oct4*), neuron (NeuN), and oligodendrocytes (Olig2). Magnification = ×200; Scale bar = 40 μm; (**B**) Immunoblots demonstrate that activation of apoptotic machinery was reduced under DD condition, which was mediated through regulation of inflammatory (*Tnfα*) and anti-oxidant molecules (Catalase, *Sod2*). L/D, 12/12-h light/dark; LL, 24-h constant light; DD, 24-h constant dark. ^a^ *p* < 0.05, vs. L/D; ^b^ *p* < 0.05, vs. LL.

Molecular analyses were performed to determine the effect of elevated melatonin during acute phase on the damage responses in the lesion site (Figure 3A). Both nestin and vimentin levels (neural stem cell markers) were increased under DD condition (^a,b^ *p* < 0.05). In addition, Ciliated ependymal cells (Nestin^+^/Vimentin^+^) have latent neural stem cell properties, which are rapidly activated to re-connect the disrupted neural circuit following spinal cord injury [8]. *Oct4* expression was also upregulated (^a,b^ *p* < 0.05) by DD condition indicating that constant darkness may enhance endogenous pluripotency following spinal cord injury. Besides, the marker of oligodendrocyte Olig2 was highly expressed in the T9-11 segments of DD group (^a,b^ *p* < 0.05). Also, the expression of NeuN (a neuronal marker) showed similar tendency (^a,b^ *p* < 0.05). It is considered that the DD condition conserved neural cells from apoptotic cell death, as compared to other groups. This was evidenced by less activation of apoptotic machinery (^a,b^ *p* < 0.05, Figure 3B), which was mediated through regulation of gene transcription (*Tnfα*, *Sod2*) and the increase of anti-oxidant catalase (^a,b^ *p* < 0.05). In contrast, LL condition exacerbated early histological severity. Spinal cord sections from all groups showed loss of ependymal lining in the central canal, scant cells mostly within the gray matter, and neutrophil degeneration in lamina IX. However, those characteristics were especially severe in the sections from LL group animals. The nestin levels between L/D and LL groups had no differences in spite of ependymal loss. Instead, LL condition induced upregulation of *Oct4* expression as well as vimentin level (^a^ *p* < 0.05). This indicates that the histological severity following constant light condition may initiate fierce remodeling since Vimentin is reported to extensively be expressed by the reactive astrocytes [26] as well as macrophages [27] besides ependymal cells [28]. No change in the GFAP level (an astrocyte marker) was found; however, *Iba1* expression (a microglial marker) was significantly increased (^a^ *p* < 0.05). Concurrently, LL condition augmented not only the level of *Tnfα* mRNA (4.3-fold) but also the Bax:Bcl-2 ratio (^a^ *p* < 0.05) suggesting vulnerability to cell death. Additionally, the LL condition appears to make the injured tissue prone to oxidative stress as antioxidant *Sod2* expression was attenuated (^a^ *p* < 0.05). Taken together, these results suggest that light/dark control might be important for the primary damage responses in the traumatic zone.

### 3.3. Constant Dark Condition Accelerates Recovery of the Rostral Region via Trk-ERK Signaling Pathway

**Figure 4:**
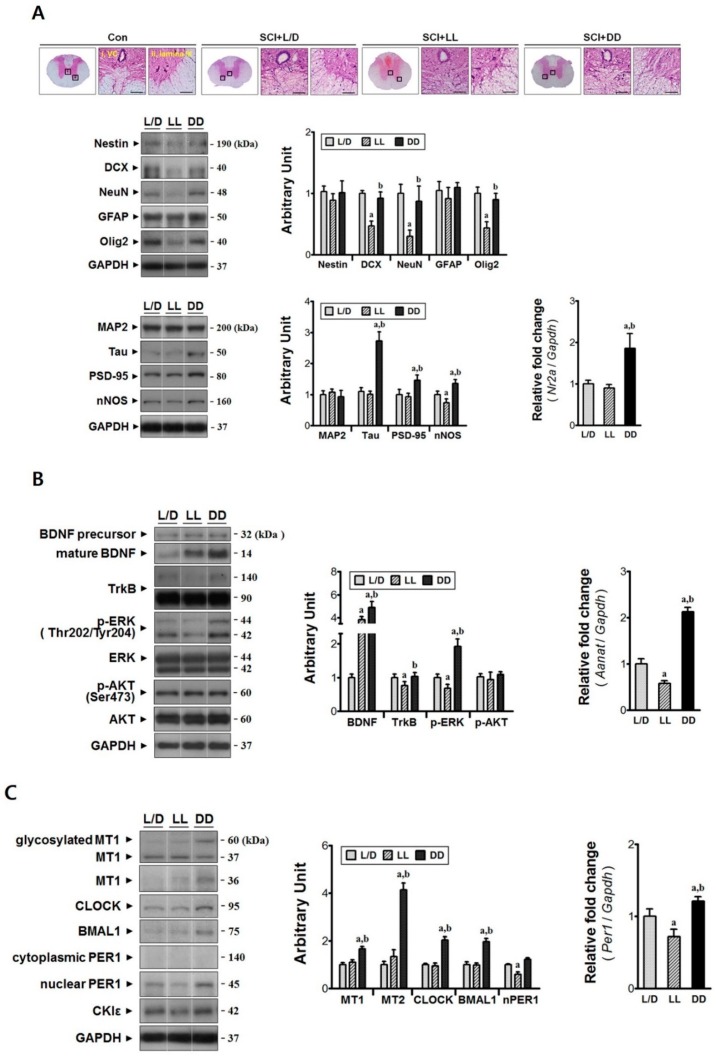
Constant dark condition accelerates excitatory synapse formation and axonal outgrowth in the rostral region via TrkB-ERK signaling pathway. The mid-thoracic segments (T6-8) were analyzed at POD 3. (**A**) The animals under LL condition showed hemorrhage in dorsal white column, interrupted neurite outgrowth, and massive loss of immature (DCX) and mature neurons (NeuN) after spinal cord injury. The DD condition promoted not only excitatory synaptic formation (PSD-95, nNOS, *Nr2a*) but also axonal outgrowth (Tau) compared with the L/D group. VC, ventral commissure; Magnification = ×200; Scale bar = 40 μm; (**B**) TrkB-mediated ERK signal transduction was enhanced under DD condition, while LL condition decreased their activation in spite of the higher level of BDNF. Transcriptional regulation of *Aanat* gene was affected by the light/dark condition; (**C**) DD condition increased the core clock proteins (CKOCK, BMAL1, nuclear PER1) as well as mRNA (*Per1*) in the rostral region. The level of melatonin receptor 1A (MT1) and 1B (MT2) was greater in the SCI + DD group than those of other groups. L/D, 12/12-h light/dark; LL, 24-h constant light; DD, 24-h constant dark. ^a^ *p* < 0.05 vs. L/D; ^b^ *p* < 0.05 vs. LL.

The mid-thoracic spinal cord (T6-8), which is the right upper segment of injury epicenter, was analyzed to determine whether light/dark condition influences the neural remodeling processes in the rostral region. Dark environment was revealed to also be beneficial to the mid-thoracic region. The level of axonal marker, Tau, was significantly augmented under DD condition (^a,b^ *p* < 0.05, Figure 4A), although neural lineage markers (Nestin, DCX, NeuN, GFAP, Olig2) were remained unchanged in the immunoblot. Moreover, this condition increased the level of PSD-95 protein (an excitatory post-synaptic marker) as well as NMDA receptor subunit *Nr2a* expression (^a,b^ *p* < 0.05). Also, Neuronal NOS (nNOS) level was also upregulated (^a,b^ *p* < 0.05), indicating that excitatory signal transduction might be activated at POD 3 in the rostral region. These changes were accompanied by regulation of TrkB and its downstream ERK signal. Exposure to DD condition did not affect total TrkB level, but facilitated ERK phosphorylation (^a,b^ *p* < 0.05, Figure 4B). TrkB receptor is known to be activated by binding of a putative ligand BDNF [19]. Interestingly, both DD and LL condition induced higher BDNF levels compared to L/D, though, TrkB-mediated ERK signal transduction was only enhanced under DD condition (^a,b^ *p* < 0.05). Moreover, exposure to DD condition enhanced the *Aanat* expression at POD 3 in the T6-8 segments (^a,b^ *p* < 0.05). This was accompanied by the increases of clock proteins (CLOCK, BMAL1, and nuclear PER1) as well as *Per1* mRNA upregulation at the time of sacrifice (^a,b^ *p* < 0.05, Figure 4C). Concurrently, the levels of melatonin receptors (MT1, MT2) were also augmented (^a,b^ *p* < 0.05). These results suggest that increase of melatonin under DD condition might induce synaptic modification through clock-controlled *Aanat* expression. In contrast, the effect of LL condition was detrimental to neural remodeling occurred in the rostral region. The neuronal lineage markers DCX, NeuN, Olig2, and nNOS protein levels were reduced (^a^ *p* < 0.05), and intraspinal hemorrhage was seen also. Exposure to LL condition suppressed neural expression of *Aanat* mRNA, TrkB glycosylation, and ERK phosphorylation (^a^ *p* < 0.05). Although NAS-TrKB-ERK signal transduction might be dampened, maturation of proBDNF was augmented (^a^ *p* < 0.05). These changes were accompanied by the reductions in *Per1* expression as well as the PER1 protein distributed in the nuclei (^a^ *p* < 0.05). Taken together, the LL condition partially inhibited the clock mechanism, and deteriorated neural remodeling in the rostral region.

### 3.4. Constant Dark Condition Preserves Excitatory Circuits in the Lumbar Region

**Figure 5:**
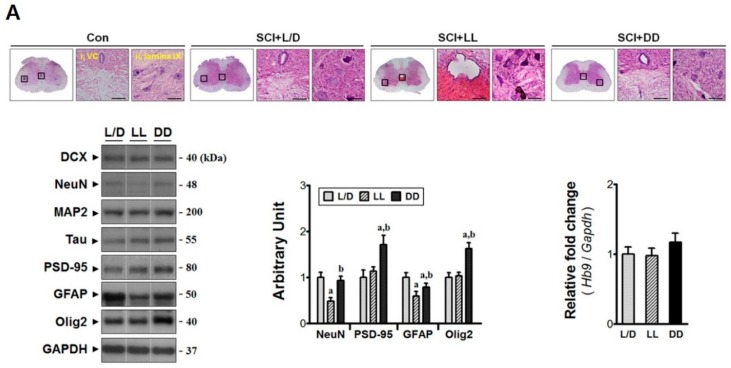

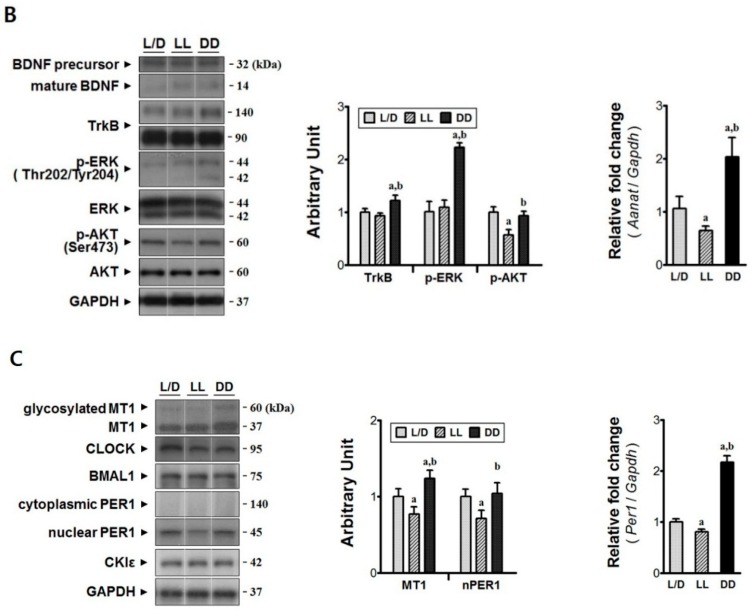
Constant dark condition preserves excitatory circuits at the acute phase in the lumbar enlargement. The spinal segments caudal to the injury epicenter (L1-2) were analyzed at POD 3. (**A**) LL condition led to the reduction of the neuroglial markers (GFAP, NeuN) as well as extensive tissue damage in the secondary lesion. Both PSD-95 and Olig2 proteins were augmented when DD condition was applied. Neuronal degeneration found in histological examination could be an evidence supporting these results. VC, ventral commissure; Magnification = ×200; Scale bar = 40 μm; (**B**) Increased *Aanat* expression under DD condition activated TrkB-ERK signal transduction, but its repression under LL condition inhibited AKT-dependent intracellular signal; (**C**) LL condition decreased *Per1* expression, nuclear distribution of PER1 protein, and glycosylated MT1 level, which was preserved under DD condition. L/D, 12/12-h light/dark; LL, 24-h constant light; DD, 24-h constant dark. ^a^ *p* < 0.05 vs. L/D; ^b^ *p* < 0.05 vs. LL.

Conditional controlled light/dark differentially regulated the neural remodeling processes in the caudal region (L1-2). All spinal cord injured rats showed dilated central canal lumen. Particularly, central canal lumen of the rats under LL condition was markedly enlarged compared to those of the rats under other conditions (Figure 5A). The ependymal lining of central canal was severely deteriorated by continuous lighting as shown in the injury epicenter, and more eosinophilic motor neurons were distributed in lamina IX. Since these “red neurons’ are considered as a hallmark of neuronal damage [29], constant light condition appears to make neuronal cells susceptible to further damage following initial injury. Also, exposure to the LL condition was revealed to decrease the population of neuronal and glial cells, and reduce neuroplastic activity, evidenced by decreased levels of NeuN, GFAP (^a^ *p* < 0.05), and phosphorylated AKT (Ser473) (^a^ *p* < 0.05, Figure 5B). Additionally, neither the total TrkB level nor ERK phosphorylation was affected, but the *Aanat* gene transcription was suppressed by LL condition (^a^ *p* < 0.05). Among clock-related molecules, *Per1* mRNA was diminished by LL condition, although the CLOCK and BMAL1 prote ins activating E-box elements in the *Per1* promotor remained unchanged (Figure 5C). In contrast, DD condition increased both *Per1* and *Aanat* expression without changes in clock proteins (CLOCK, BMAL1, nuclear PER1). In turn, upregulated *Aanat* expression activated TrkB-ERK signaling pathway that augmented the levels of PSD-95 and Olig2 protein in the lumbar spinal cord.

### 3.5. The Influences of Light/Dark Environment on the Recovery of Injured Cord Evidenced by Histological Examination

**Figure 6:**
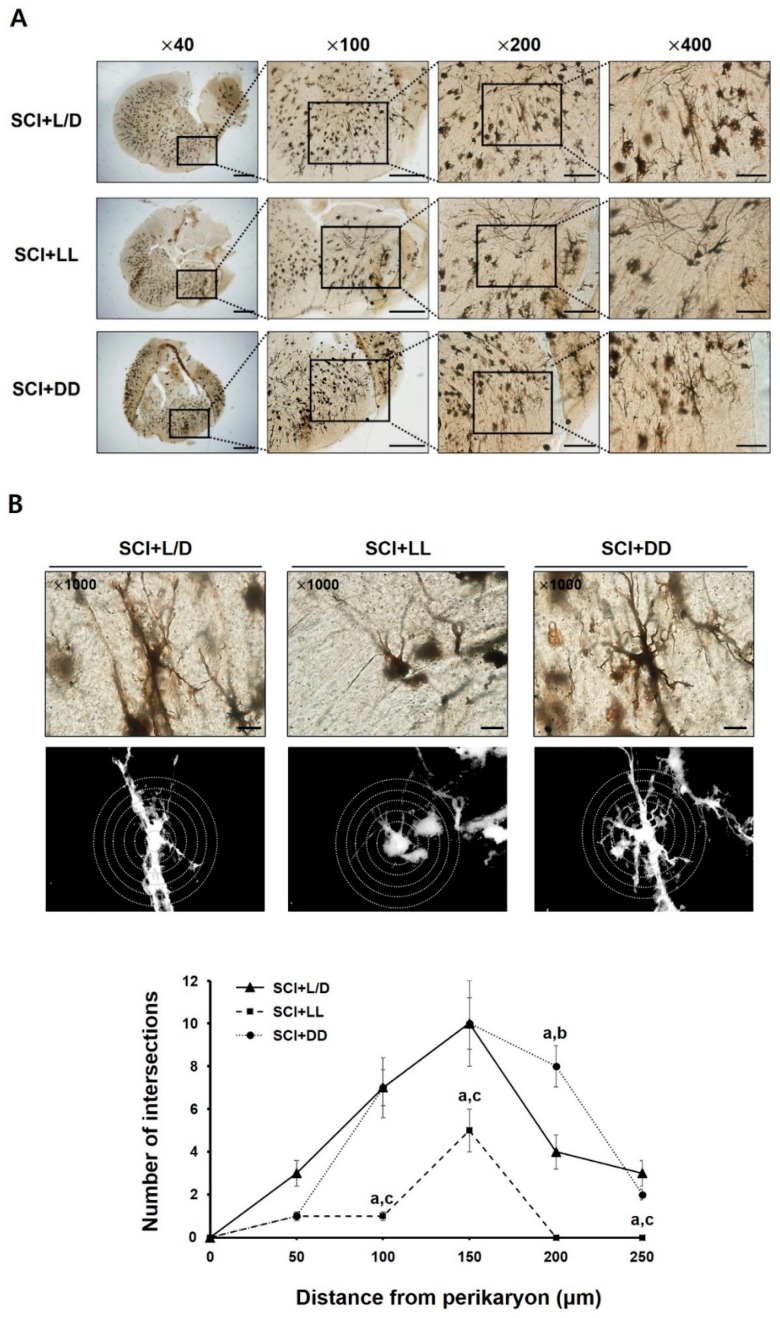
Golgi-cox stained sections of spinal cord from the rats subjected to controlled light/dark cycle. (**A**) Experimental animals were kept in controlled light/dark cycle; L/D, LL, and DD. A total of 3 days after SCI surgery, histological differences in the injured spinal segment were investigated. Magnification = ×4, ×100, ×200, ×400; Scale bar = 500 μm (×40, ×100), 200 μm (×200, ×400). (**B**) Neurite outgrowth of the neurons in Golgi-stained spinal sections was quantified using Sholl analysis method. The Sholl rings are separated by 10 μm. The data were expressed as mean ± SD. L/D, 12/12-h light/dark; LL, 24-h constant light; DD, 24-h constant dark. ^a^ *p* < 0.05 vs. L/D; ^b^ *p* < 0.05 vs. LL; Magnification = ×1000; Scale bar = 100 μm.

Conditional controlled light/dark cycles also may induce histological alterations in cord lesion. The spinal cord lesion of the rats under DD condition showed a comparatively dense population of neural cells; whereas the LL condition resulted in low density of neural cells on injured segment (Figure 6A). Specifically, it has been revealed by the comparison of neurite outgrowth, using Sholl analysis, that rats under the LL condition showed sparse intersections (neurite outgrowth) in the injured spinal segment compared with other two groups. Namely, injured spinal segment of the rats under LL condition showed fewer neural intersections in its anterior horn region within the radius of 250 μm apart from perikaryon. In addition, the cord of the rats under DD condition showed abundant neuronal intersections in the anterior horn at the point 200 μm apart from perikaryon, compared with other groups. In general, SCI + LL group showed poor histological recovery among the groups (Figure 6B). These results represent that light/dark environment can influence the neuroplastic mechanisms following traumatic SCI, the regulation of which might be mediated via TrkB-AKT/ERK signal transduction.

## 4. Discussion

Evidences from several studies have suggested that acute clinical management is important to prevent secondary complications and promote functional recovery [30,31]. However, an inappropriate environment in the hospital might act as a risk factor that exacerbates the pathological processes. For example, artificial bright light might be detrimental because environmental light/dark influences melatonin-generating machineries in various organisms [32,33,34]. Besides secretory regulation, light/dark cycle differentially regulates the density and distribution of melatonin receptor MT1 [35]. Melatonin protects the neuronal structures from injury [12], which is based on anti-inflammatory [18] as well as anti-oxidative capacity [36]. Supplementary administration promotes neurogenesis through both the proliferation and differentiation of neural stem cells [11,37,38]. These suggest that decreased melatonin availability would be disadvantageous to the repair processes following neural damage. Moreover, melatonin is capable of re-synchronizing circadian rhythm disrupted by constant light [39]. Light-induced circadian disruption caused malignant progression via an Wnt signaling [40]. These compelled us to examine the effects of endogenous melatonin controlled by the light/dark condition on neural recovery after spinal cord injury.

It has been documented by several researchers that endogenous melatonin levels may be altered by CNS injury, including SCI. Hulten et al. [41] insisted that cervical SCI patients have reduced melatonin secretion compared to that of thoracic SCI patients. However, as subjects of the study performed by Hulten and colleagues had chronic SCI, the results might not coincide with our study which was conducted with acute SCI rats. Many more studies have implied increased endogenous melatonin following detrimental conditions. Stress condition was proved to elevate CSF melatonin metabolites [42]. Namely, pineal function appears to be regulated by physiological stress [43]. Similarly, it was reported that the concentration of endogenous melatonin was elevated in the CSF of humans with post-traumatic brain injury (TBI) [44], which was a compensatory response to attenuate oxidative stress and/or inflammation following the damage. Taken together, as one of the stress conditions and CNS injury, SCI itself contributes to increase in endogenous melatonin levels. In our results, the level of CSF melatonin increased in all of the acute spinal cord injured animals. This phenomenon might be resulted from anti-damage demands after spinal cord injury, even though they were exposed to LL condition. However, LL condition disrupted local clock machinery with *Aanat* suppression in the entire spinal cord. *Aanat* gene is known to encode the clock-driven enzyme to synthesize NAS leading to the activation of circadian-dependent TrkB [19,20]. In turn, TrkB-Akt/ERK signal transduction may engage in neural remodeling [21]. In this context, NAS-mediated neuroprotection is thought to be inhibited by LL condition. This constant light condition is improper for inducing an early neural remodeling, making it hard for the injured cord to be recovered in a short period. 

In contrast, DD condition augmented the MT1 level as well as the concentration of melatonin, indicating relatively high melatonin availability at acute phase. Melatonin receptor MT1 belongs to the G-protein-coupled receptor (GPCR) superfamily of membrane receptors [45]. MT1 can be potentially glycosylated at their extracellular N-termini [46]. In our results, rats under DD condition demonstrated elevated expression of melatonin receptor MT1 when compared with that under natural light/dark cycle. This might be associated with increased MT1 glycosylation under constant dark condition. Although a clear role for the glycosylated melatonin receptors has not yet been elucidated, it might be associated with increased stability at the plasma membrane, as for many other GPCRs [47]. The function of SCN is rhythmically entrained by melatonin through regulation of MT1 [48]. And MT2 signaling determines the phase of circadian clock located in SCN [49,50]. Moreover, CLOCK:BMAL1 heterodimers can be bound to the non-canonical E-box in MT2 promoter region, leading to initiation of transcription itself [51]. These indicate that regulation of clock machinery can be mediated by melatonin receptors. In the present study, elevated melatonin under DD condition augments the level of melatonin receptors in thoracic region rostrally from the injury epicenter. Additionally, it promoted not only transcription of core clock gene *Per1* but also clock-controlled *Aanat* expression in both rostral and caudal region. Although we are unsure whether the time-dependent rhythmicity was altered, DD condition, at least early post-traumatic days, might influence the clock machinery mediated by melatonin receptors in the penumbra. Especially, *Aanat* gene encodes the clock-driven enzyme to synthesize NAS that induces circadian-dependent TrkB activation [19,20]. Because TrkB-Akt/ERK signal transduction is involved in the neural remodeling processes, DD condition might facilitate the neural repair at least ZT13-15. Melatonin modulates the pro-survival mechanism via TrkB-induced Akt/ERK signaling [21]. Therefore, we suggest that environmental light/dark control might be critical in the early repair processes. 

In the present study, we used anatomically incomplete SCI animals, and observed injury-induced neural remodeling throughout the entire spinal cord. The majority of researches in preclinical neuroplasticity has used incompletely injured SCI animals [52], which is the most frequent type of SCI in humans [53]. Interventional approaches that aim to facilitate neural repair of large lesions have been considered unrealizable for many decades; however, recent studies have suggested that the CNS has a high neuroplastic capacity to restore complex motor behaviors after injury. For example, rhythmic locomotion, which is a representative complex behavior, is produced by central pattern generators (CPGs). Glutamatergic interneurons, elementary components of CPGs, are necessary for rhythm generation. Glutamatergic reticulospinal neurons as well as serotonergic neurons located in the hindbrain are thought to be CPGs that mediate the descending locomotor command to the spinal cord [54]. In adult higher vertebrates, the long-distance connection of axotomized supraspinal fibers is exhaustively regulated by growth-inhibitory substances. The spared neuronal fibers try to re-innervate the adjacent, denervated spinal targets, forming a *de novo* spinal circuit [52]. Vesicular glutamate transporters (vGlut) are expressed in the synaptic terminals of excitatory supraspinal and intraspinal neurons. Both vGlut1- and vGlut2-positive immunoreactivities are reduced in the subacute phase (1 week post-injury) in the caudal region from the injury epicenter [55]. This indicates a reduction in excitatory neurotransmission in the lumbar enlargement [10,55], which might be involved in early locomotor defects such as dragging hind legs in injured animals. In our results, DD condition augmented the level of PSD-95 protein at POD 3 compared to the different condition. This indicates that excitatory synapses might be constructed by 24-h dark in the acute phase because PSD-95 protein is a major scaffolding molecule that is enriched at glutamatergic synapses [56]. 

In addition to synaptic remodeling, the fate of immature neurons was also regulated by conditional light control. LL condition diminished the level of DCX, NeuN, and Olig2 in the adjacent rostral region. Histologically, massive injury was observed in the adjacent rostrocaudal area. In particular, the central canal was more severely deteriorated, indicating a low regenerative potential of endogenous stem cells [8]. Reactive gliosis, which mainly appears in the acute stage [57], secretes both growth-stimulating and growth-inhibitory factors after SCI. However, the production of inhibitory components surpasses the growth-promoting factors [58]. Thus, delayed reactive gliosis in the lumbar region might be detrimental to neural recovery because of the formation of posttraumatic glial scars [59]. Therefore, we suggest that intentional dark induces early, better, and favorable outcomes, which might be resulted from beneficial changes of neuroplasticity mediated by augmented endogenous melatonin. 

In this study, we have suggested beneficial roles of endogenous melatonin. However, some limitations exist. Although this study elucidated the beneficial influences of endogenous melatonin, the experimental DD condition could not directly be applied to human subjects as the condition may accompany negative psychological responses. To overcome this limitation, exogenous melatonin treatment could be an alternative way. Indeed, our lab has previously reported that endogenous and exogenous melatonin can contribute to the neural recovery after SCI [18]. Therefore, based on our results, further studies would be needed to find the optimal way to translate these studies to human subjects. Although we did not measure the melatonin level in the different groups of animals after 2 weeks of pre-adaptation to controlled light cycles, this limitation is overcome by several previous studies. It has been reported that exposure to constant light causes a decrease in melatonin levels in rats followed by changes in metabolism, and circadian rhythms [60]. Likewise, chronic light exposure was proved to suppress melatonin secretion in human [61]. More recently, long-term constant darkness was revealed to increase serum melatonin concentration [62]. These studies insist that light condition apparently has influence on endogenous melatonin levels, and could be the explanation to our limitation. In this context, each group was considered to have different melatonin levels after 2 weeks of pre-adaptation before being subjected to SCI surgery, depending on the pre-adaptation to each light condition. 

## 5. Conclusions

**Figure 7:**
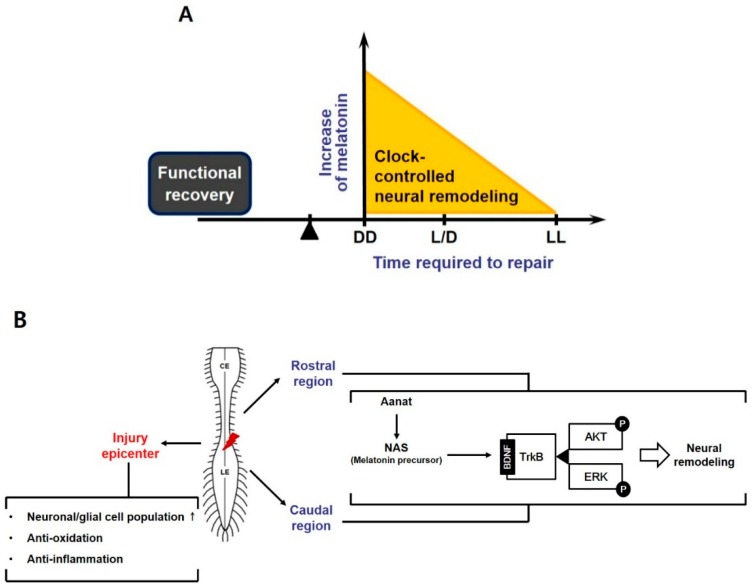
Schematic diagram summarizing our results. (**A**) Different light/dark conditions influenced the level of endogenous melatonin distributed in CSF, which might regulate the clock-driven neural remodeling processes at the acute phase. Time required for neural repair might be shortened under DD condition, but LL condition delayed the recovery speed; (**B**) Early elevated endogenous melatonin might act on the rostral (T6-8) and caudal penumbra (L1-2) as well as the injury epicenter (T9-11). Increased melatonin secretion under DD condition elevated neuronal and glial population, and exerted the anti-inflammatory and anti-oxidative actions, which might have protective role against primary cell death in the epicenter. Early dark condition enhanced clock-controlled *Aanat* expression in the rostral and caudal penumbra, leading to activation of NAS-TrkB-AKT/ERK signal transduction pathway. These might influence post-SCI neural remodeling including excitatory neurotransmission as well as axonal sprouting, followed by advance in motor recovery, ultimately.

In this study, we discovered that manipulation of melatonin synthesis through environmental light/dark cycle control may affect the recovery after SCI (Figure 7A). We observed relatively high endogenous melatonin concentration in the CSF of the animals under DD condition. This high level of melatonin seems to play anti-inflammatory and anti-oxidative roles in injured spinal segments leading to early neural remodeling including oligodendrogenesis, excitatory synaptic formation, and axonal outgrowth mediated through NAS-TrkB-AKT/ERK signal transduction (Figure 7B). Moreover, abundant melatonin may give rise to the acceleration of excitatory synapse formation and axonal outgrowth as well as preservation of excitatory circuits in the rostral (T6-8) region. In conclusion, these findings suggest that endogenous melatonin, which is regulated by light/dark control, is a crucial factor that affects neural repair after SCI.

## Figures and Tables

**Table 1:** Oligonucleotide primers used for PCR.

Gene	Primer Sequences (5′-3′)	Size (bp)	GenBank Accession No.
*Aanat*	F: AAA GTA CAC TCA GGC ACC AAT GT	110	NM_012818
	R: GGG AAC ATA GCT GCT TTA TTA GTG TCA G		
*Hb9*	F: GCA ATC GAA CCT CTT GGG GA	187	NM_001271274.1
	R: TTT CAT TCG GCG GTT CTG GA		
*Iba1*	F: GTC CTT GAA GCG AAT GCT GG	157	NM_017196.3
	R: CAT TCT CAA GAT GGC AGA TC		
*Nr2a*	F: TCC ATT CTT CTG TCA TCC TGC	224	NM_012573.3
	R: AAG ACC GTC TCT CAC TCT TGC		
*Oct4*	F: GAG GGA TGG CAT ACT GTG GAC	272	XM_228354
	R: GGT GTA CCC CAA GGT GAT CC		
*Per1*	F: TTT GGA GAG CTG CAA CAT TCC	101	NM_011065.4
	R: CTG CCC TCT GCT TGT CAT CA		
*Sod2*	F: CCG AGG AGA AGT ACC ACG AG	174	NM_017051.2
	R: GCT TGA TAG CCT CCA GCA AC		
*Tnfα*	F: CTA CTG AAC TTC GGG GTG ATC	292	NM_012675.3
	R: CTT GTC CCT TGA AGA GAA CCT G		
*Gapdh*	F: CTC AGT TGC TGA GGA GTC CC	120	NM_017008.4
	R: ATT CGA GAG AAG GGA GGG CT		
